# Circulating Th1, Th2, and Th17 Levels in Hypertensive Patients

**DOI:** 10.1155/2017/7146290

**Published:** 2017-07-05

**Authors:** Qingwei Ji, Guojie Cheng, Ning Ma, Ying Huang, Yingzhong Lin, Qi Zhou, Bin Que, Jianzeng Dong, Yujie Zhou, Shaoping Nie

**Affiliations:** ^1^Emergency & Critical Care Center, Beijing Anzhen Hospital, Capital Medical University, and Beijing Institute of Heart, Lung, and Blood Vessel Diseases, Beijing 100029, China; ^2^Department of Cardiology, Beijing Anzhen Hospital, Capital Medical University, and Beijing Institute of Heart, Lung, and Blood Vessel Diseases, The Key Laboratory of Remodeling-Related Cardiovascular Disease, Ministry of Education, Beijing 100029, China; ^3^Department of Cardiology, The People's Hospital of Guangxi Zhuang Autonomous Region, Nanning 530021, China; ^4^Department of Ultrasound, The People's Hospital of Guangxi Zhuang Autonomous Region, Nanning 530021, China

## Abstract

**Background:**

Evidence from experimental studies showed that Th1, Th2, and Th17 play a pivotal role in hypertension and target organ damage. However, whether changes in the circulating Th1, Th2, and Th17 levels are associated with nondipper hypertension and carotid atherosclerotic plaque in hypertension has yet to be investigated.

**Methods:**

Th1, Th2, and Th17 levels were detected using a flow cytometric analysis, and their related cytokines were measured by enzyme-linked immunosorbent assay in 45 hypertensive patients and 15 normotensive subjects.

**Results:**

The frequencies of Th1 and Th17 in hypertensive patients, especially in nondipper patients and patients with carotid atherosclerotic plaque, were markedly higher than those in the control group; this was accompanied by higher IFN-*γ* and IL-17 levels. In contrast, the Th2 frequencies and IL-4 levels in hypertensive patients, especially in nondipper patients and patients with carotid atherosclerotic plaque, were significantly lower than those in the control group.

**Conclusions:**

The changes in Th1, Th2, and Th17 activity are associated with the onset of the nondipper type and carotid atherosclerotic plaque in hypertensive patients.

## 1. Introduction

Hypertension is a clinical syndrome defined as systolic blood pressure (SBP) levels in excess of 140 mm Hg or diastolic blood pressure (DBP) levels greater than 90 mm Hg. Epidemiological evidence demonstrated that sustained uncontrolled high blood pressure leads to target organ damage, eventually exacerbating the occurrence of cardiovascular events, including atherosclerotic disease, heart failure, and aortic dissection. According to the ambulatory blood pressure monitoring (ABPM), hypertension can be divided into two types: nondipper hypertension and dipper hypertension. Dipper hypertension is defined as a drop of 10% or more in blood pressure values of night-time than daytime whereas nondipper hypertension is defined as a drop of less than 10% in blood pressure values of night-time than daytime [1]. Previous studies showed that ambulatory blood pressure can predict mortality better than clinic blood pressure, and dippers have lower all-cause mortality than nondippers [2–4].

CD4+ effector T (Teff) cells play a critical role in cardiovascular disease, including atherosclerosis, hypertension, and heart failure [5–9]. According to their cytokine secretion profile, Teff cells are functionally divided into three subsets: Th1, Th2, and Th17. Some studies indicated that the Th1 immune response is associated with blood pressure elevation and enlarged atherosclerotic size [5]. Our previous study demonstrated that Th2 response was suppressed by exogenous angiotensin II in a hypertensive hypercholesterolemic model and played a critical role in the antiatherosclerotic effects of valsartan, an AT1 receptor blocker (ARB) [6]. However, we found that the Th2 response has no effect on blood pressure values in that model [6]. Interestingly, Madhur et al. found that blocking the Th17 response resulted in a reduction in blood pressure but had no effect on atherosclerotic lesion size [7]. Evidence from clinical studies has revealed that changes in Th1, Th2, and Th17 responses are associated with the occurrence of pregnancy-induced hypertension, which is a special type of hypertension [10]. Another study reported that an overactive Th17 immune response exists in hypertensive patients with carotid plaque and could be attenuated by telmisartan and rosuvastatin treatment [11]. However, because data have identified that changes in the Th1, Th2, and Th17 responses exist in atherosclerotic patients with hypertension [11], it is difficult to recognize whether the change in Teff cell activity is associated with hypertension. In addition, many studies suggest that changes in serum levels of IFN-*γ*, IL-4, and IL-17, which are the characteristic factor of Th1, Th2, and Th17, respectively, may play a role in hypertension [12–14]. Therefore, we investigated the Th1, Th2, and Th17 type responses in 45 patients with primary hypertension in the present study. We also aimed to determine the relationship of the circulating Th1, Th2, and Th17 levels with nondipper hypertension and carotid atherosclerosis in these patients.

## 2. Materials and Methods

Forty-five patients with primary hypertension and 15 healthy subjects were enrolled in the present study. All of the patients were examined by ABPM and B-mode ultrasound examinations. B-mode ultrasound was performed on all of the patients to identify whether they have carotid atherosclerosis. The ultrasonography was performed by trained, certified sonographers with a Philips iU22 device and a linear transducer of 8-9 MHz according to a standardized protocol [15]. In brief, both vertical and transverse scanning along the carotid arteries were performed when the patient lay in supine position, with his head turned to the opposite side. Carotid plaque assessment was performed as recommended by the Manheim Carotid Intima-Media Thickness and the American Society of Echocardiography Consensus. The presence of plaque (yes/no) on either of the two sides in the common and internal carotid arteries was defined as a focal thickening (≥1.5 mm) into the lumen on gray-scale imaging. Reproducibility was verified in 10 randomly selected patients scanned twice on each side (20 arteries in total), and the variation coefficient was estimated to be 8.5%. The ABPM and B-mode ultrasound examinations confirmed that the healthy control subjects had normal blood pressure, a dipper profile, and noncarotid atherosclerosis. The study conformed to the guidelines approved by the ethics committee at our institution.

There were no patients with secondary hypertension, coronary artery disease, heart failure, stroke, valvular heart disease, collagen disease, advanced liver disease, renal failure, malignant disease, septicemia, or other inflammatory diseases.

Blood samples were obtained the morning following admission from fasted patients in a recumbent position with a 21-gauge needle for a clean venipuncture of an antecubital vein. Samples were collected into sodium heparin vacutainers (Becton-Dickinson). Peripheral blood mononuclear cells (PBMCs) were prepared in a Ficoll density gradient for analysis via flow cytometry. Plasma obtained after centrifugation was stored at −80 until further use.

PBMCs were suspended at a density of 2.0 × 10^6^ cells/mL in complete culture medium (Gibco BRL, USA). These cells were treated for 4 h with the following stimuli: 25 ng/mL phorbol myristate acetate (PMA, Alexis Biochemicals, San Diego, CA) and 1 *μ*g/mL ionomycin (Alexis Biochemicals, San Diego, CA) in the presence of 1 *μ*L of monensin (Ebioscience, San Diego, CA) after culturing. The contents of the well were transferred to 12 × 75 mm polypropylene tubes. The cells were then centrifuged at 1500 rpm for 5 min. Then, cells were aliquoted into three tubes and washed twice with phosphate-buffered saline (PBS). For Th1 and Th17 analysis, the cells were incubated with PE-CY7 anti-human CD4 for 20 min at room temperature. After fixation and permeabilization, according to the manufacturer's procedures, the cells were incubated for 20 min with FITC anti-human IFN-*γ* and PE anti-human IL-17A for Th1 (CD4+ IFN-*γ*+ IL-17−/CD4+ T cells) and Th17 (CD4+ IFN-*γ*− IL-17+/CD4+ T cells) detection. For Th2 analysis, the cells were incubated with PE-CY7 anti-human CD4 for 20 min at room temperature. After fixation and permeabilization, according to the manufacturer's procedures, the cells were incubated for 20 min with PE anti-human IL-4 for Th2 (CD4+ IL-4+/CD4+ T cells) detection. Finally, the cells were analyzed by flow cytometric analysis using a FACS Aria cytometer (BD Biosciences, San Jose, CA).

The levels of IFN-*γ*, IL-4, and IL-17 were measured with an enzyme-linked immunosorbent assay (ELISA) following the manufacturer's instructions (Bender MedSystems, Burlingame, California). The minimal detectable concentrations were 4 pg/mL for IFN-*γ*, 0.4 pg/mL for IL-4, and 2 pg/mL for IL-17. All assays were performed in duplicate.

All of the data are presented as the means ± SD. The data were analyzed using SPSS 17.0 statistical software (LEAD Technologies Inc., Chicago, IL, USA). Student's *t*-test was used when comparing only 2 groups. For comparisons involving 3 groups, one-way ANOVA followed by Neuman-Keuls post hoc test was used. Pearson's correlation was used to calculate the correlations between Th1, Th2, and Th17 activities and the other measured parameters. Simple linear regression analyses and subsequent binary logistic regression analyses were performed to identify the independent predictors of the presence of nondipper hypertension and CAP. The candidate variables entered in the model included age; sex; body mass index (BMI); heart rate; lipid and lipoprotein fractions; fasting glucose (GLU); hemoglobin A1c (HbA1c); creatinine; C-reactive protein (CRP); homocysteine (Hcy); angiotensin II; Th1, Th2, and Th17 frequencies; and the levels of IFN-*γ*, IL-4, and IL-17. In all of the tests, a value of *P* < 0.05 was considered to be statistically significant.

## 3. Results

There were no significant differences in age, sex, heart rate, total triglycerides (TG), total cholesterol (TC), low-density lipoprotein cholesterol (LDL-C), GLU, HbA1c, or creatinine between the control and hypertension groups (Table 1). The office blood pressure, BMI, CRP, Hcy, and angiotensin II levels were significantly higher in the hypertension group than in the control group, whereas the high-density lipoprotein cholesterol (HDL-C) levels were significantly lower in the hypertension group than in the control group.

According to the results of B-mode ultrasound examinations, 7 patients with dipper hypertension and 10 patients with nondipper hypertension had carotid atherosclerotic plaque (CAP group), and 28 hypertensive patients had no carotid atherosclerotic plaque (NCAP group). There were no significant differences in sex, heart rate, BMI, lipid and lipoprotein fractions, GLU, HbA1c, creatinine, CRP, Hcy, or angiotensin II between the CAP and NCAP groups (Table 1). The office DBP levels were significantly higher in the NCAP group than in the CAP group, whereas the age and year of hypertension were significantly higher in the CAP group than in the NCAP group.

According to the results of ABPM, the hypertension patients were divided into the following two groups: 20 patients with dipper hypertension and 25 patients with nondipper hypertension. There were no significant differences in age, sex, heart rate, BMI, office blood pressure, lipid and lipoprotein fractions, GLU, HbA1c, creatinine, CRP, Hcy, or angiotensin II between the dipper and nondipper patients (Table 1). The average 24 h SBP and DBP, the average daytime SBP and DBP, and the average night-time SBP and DBP were significantly higher in hypertensive patients, including the dipper and nondipper patients and the CAP and NCAP patients, than in the control group (Table 2). The results showed that the average 24 h SBP and DBP, the average daytime SBP and DBP, and the average night-time DBP were significantly lower in the CAP patients than in the NCAP group. However, there were no differences in the average 24 h SBP and DBP, the average daytime SBP and DBP, and the average night-time SBP and DBP between the dipper and nondipper patients.

**Figure 1:**
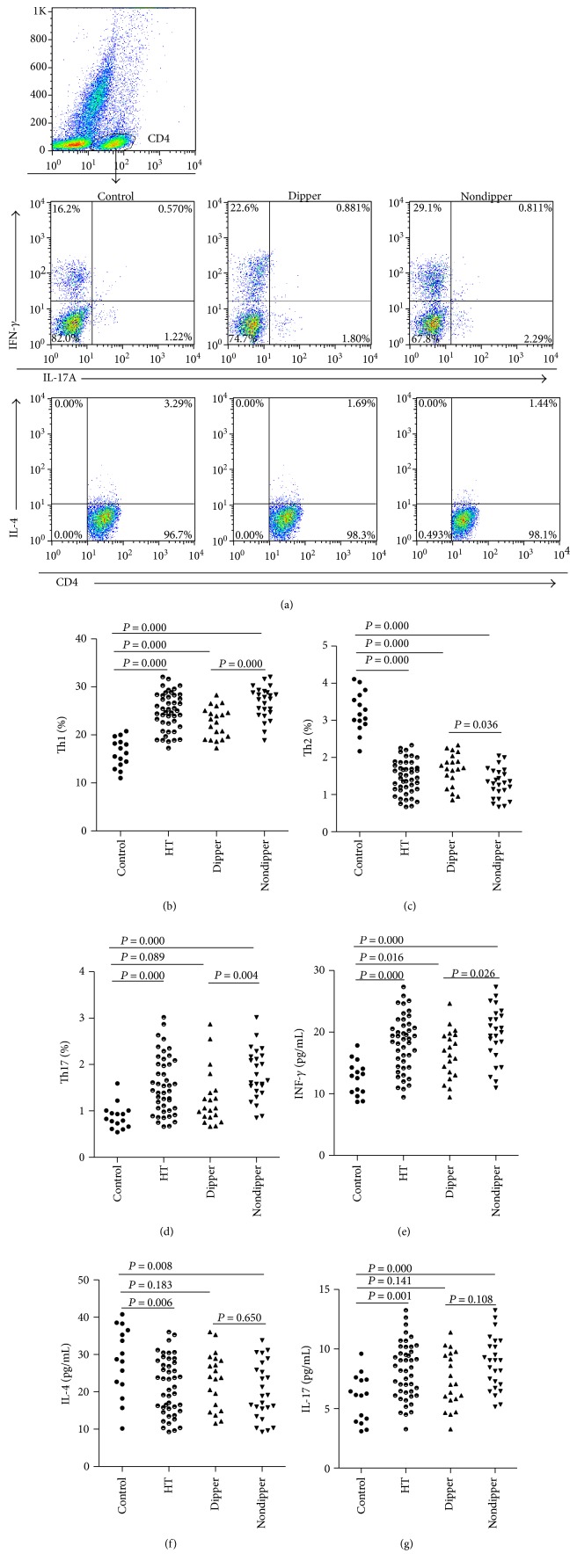
Circulating Th1, Th2, and Th17 levels in the dippers and nondippers. (a) CD4+ T cells were gated by flow cytometry and representation of intracellular cytokine staining of Th1, Th2, and Th17 from each group. (b) The frequencies of Th1 were markedly higher in hypertensive patients, including the dippers and nondippers, than in the control group. (c) The frequencies of Th2 were markedly lower in hypertensive patients, including the dippers and nondippers, than in the control group. (d) The frequencies of Th17 were markedly higher in hypertensive patients, especially in nondippers, than in the control group. (e) IFN-*γ* levels in the hypertension group, including dipper and nondipper patients, were markedly higher than those in the control group. (f) IL-4 levels in the hypertension group, especially in nondipper patients, were markedly lower than those in the control group. (g) IL-17 levels in the hypertension group, including dipper and nondipper patients, were markedly higher than those in the control group.

As shown in Figure 1 and Table 3, the frequency of Th1 in the hypertension group, including the dipper and nondipper patients, was markedly higher than that in the control group. The frequency of Th1 in the nondipper patients was markedly higher than that in the dipper patients. The frequency of Th2 in the hypertension group, including dipper and nondipper patients, was markedly lower than that in the control group. Additionally, the frequency of Th2 in the nondipper patients was markedly lower than that in the dipper patients. The frequency of Th17 in the hypertension group, especially among the nondipper patients, was markedly higher than that in the control group. In addition, the frequency of Th17 among the nondipper patients was markedly higher than that among the dipper patients.

**Figure 2:**
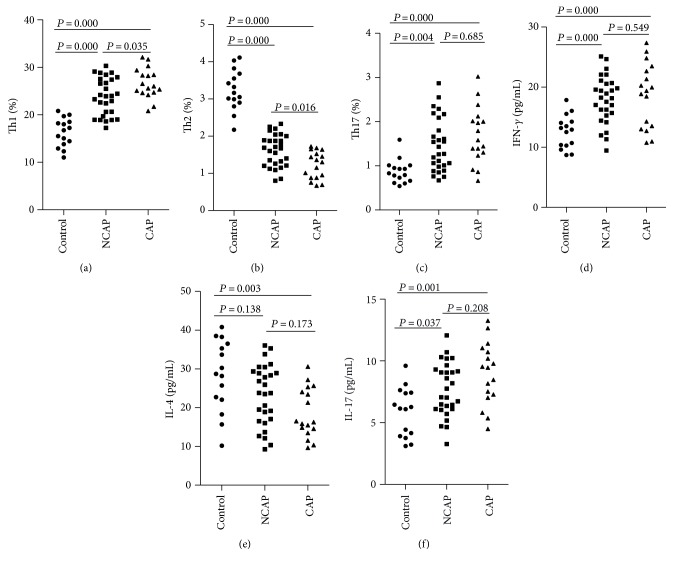
Circulating Th1, Th2, and Th17 levels in hypertensive patients with or without CAP. (a) The frequency of Th1 in the NCAP and CAP patients was markedly higher than that in the control group. (b) The frequency of Th2 in the NCAP and CAP patients was markedly lower than that in the control group. (c) The frequency of Th17 in the NCAP and CAP patients was markedly higher than that in the control group. (d) IFN-*γ* levels in the NCAP and CAP patients were markedly higher than that in the control group. (e) IL-4 levels in the CAP patients were markedly lower than that in the control group. (f) IL-17 levels in the NCAP and CAP patients were markedly higher than that in the control group.

We measured Th1, Th2, and Th17 levels in hypertensive patients with or without CAP. As shown in Figure 2 and Table 3, the frequency of Th1 in the NCAP and CAP patients was markedly higher than that in the control group, while the frequency of Th1 in the CAP patients was markedly higher than that in the NCAP patients. The frequency of Th2 in the NCAP and CAP patients was markedly lower than that in the control group, while the frequency of Th2 in the CAP patients was markedly lower than that in the NCAP patients. The frequency of Th17 in the NCAP and CAP patients was markedly higher than that in the control group, whereas there were no differences between the NCAP and CAP patients.

We also investigated whether sex and medication use affected the levels of Th1, Th2, and Th17 in hypertensive patients. The results showed that neither sex nor the administration of angiotensin-converting enzyme inhibitors (ACEIs) or angiotensin receptor blockers (ARBs), *β*-blockers, or calcium channel blockers (CCBs) had significant effects on Th1, Th2, and Th17 levels (Table 4). Only the administration of diuretics was associated with lower Th2 levels and higher Th17 levels in hypertensive patients.

As shown in Figure 1 and Table 3, IFN-*γ* levels in the hypertension group, including dipper and nondipper patients, were markedly higher than those in the control group. Additionally, IFN-*γ* levels in nondipper patients were markedly higher than those in dipper patients. IL-4 levels in the hypertension group, especially in nondipper patients, were markedly lower than those in the control group. However, there were no differences in the IL-4 levels between the dipper and nondipper patients. IL-17 levels in the hypertension group, including dipper and nondipper patients, were markedly higher than those in the control group. Additionally, IL-17 levels in nondipper patients were markedly higher than those in dipper patients.

We also measured the levels of the three cytokines in hypertensive patients with or without CAP. As shown in Figure 2 and Table 3, IFN-*γ* and IL-17 levels in the NCAP and CAP patients were markedly higher than those in the control group, whereas there were no differences between the NCAP and CAP patients. In contrast, IL-4 levels in the CAP patients were markedly lower than those in the control group, whereas there were no differences between the NCAP and CAP patients.

We next investigated whether sex and medication use affected IFN-*γ*, IL-4, and IL-17 concentrations in hypertensive patients. The results showed that neither sex nor the administration of ACEI (ARB), CCB, or diuretics had significant effects on IFN-*γ*, IL-4, and IL-17 levels (Table 4). Only the administration of *β*-blocker was associated with an increase in IL-17 levels in hypertensive patients.

**Figure 3:**
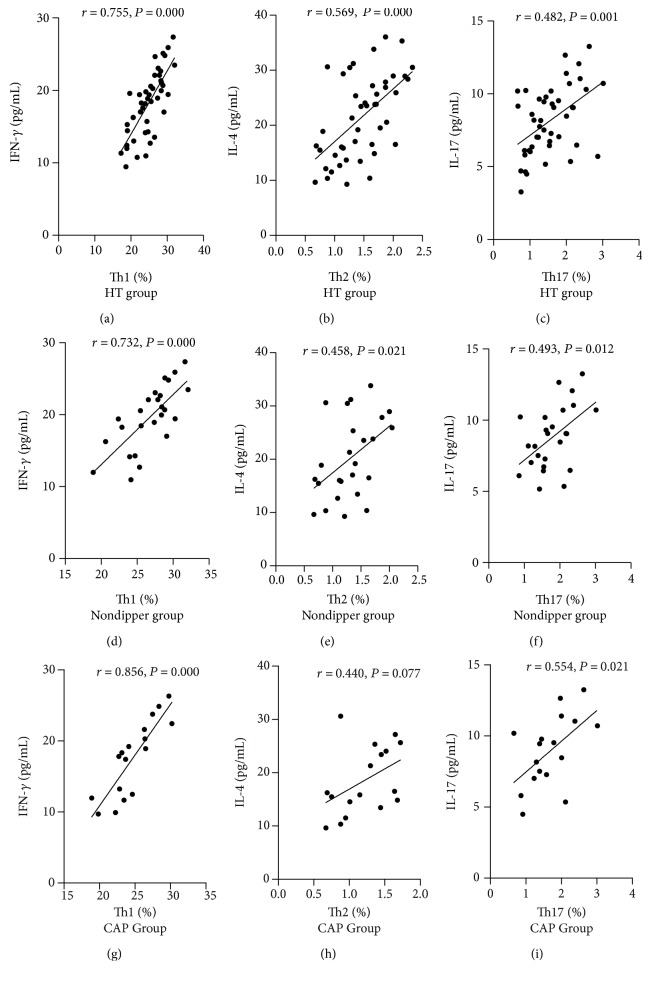
Correlation analysis of circulating Th1, Th2, and Th17 levels with IFN-*γ*, IL-4, and IL-17 concentrations in hypertensive patients, nondipper patients, and CAP patients. (a) The frequencies of Th1 cells showed a positive correlation with IFN-*γ* levels in hypertensive patients. (b) The frequencies of Th2 cells showed a positive correlation with IL-4 levels in hypertensive patients. (c) The frequencies of Th17 cells showed a positive correlation with IL-17 levels in hypertensive patients. (d) The frequencies of Th1 cells showed a positive correlation with IFN-*γ* levels in nondippers. (e) The frequencies of Th2 cells showed a positive correlation with IL-4 levels in nondippers. (f) The frequencies of Th17 cells showed a positive correlation with IL-17 levels in nondippers. (g) The frequencies of Th1 cells showed a positive correlation with IFN-*γ* levels in CAP patients. (h) There were no significant correlations between the frequencies of Th2 cells and IL-4 levels in CAP patients. (i) The frequencies of Th17 cells showed a positive correlation with IL-17 levels in CAP patients.

We analyzed the correlations of circulating Th1, Th2, and Th17 levels with IFN-*γ*, IL-4, and IL-17 concentrations in hypertensive patients, nondipper patients, and CAP patients. The results showed that circulating levels of Th1 and Th17 positively correlated with IFN-*γ* and IL-17 levels, respectively, in hypertensive patients, nondipper patients, and CAP patients (Figure 3). The positive correlations were also observed between circulating Th2 and IL-4 levels in hypertensive patients and nondipper patients but not in CAP patients.

We also analyzed the correlations of circulating Th1, Th2, and Th17 levels with blood pressure in hypertensive patients, nondipper patients, and CAP patients. The results showed that the frequencies of Th1, Th2, and Th17 had no significant correlations with blood pressure in hypertensive patients, nondipper patients, and CAP (Table 5). However, the correlation analysis showed that IFN-*γ* levels were positively correlated with average night-time SBP in hypertensive patients (Table 5). Moreover, IL-17 levels were negatively correlated with average daytime DBP in hypertensive patients and DBP in nondipper patients (Table 5).

Then, we assessed whether the changes in circulating Th1, Th2, and Th17 levels were associated with age, years of hypertension, heart rate, body mass index (BMI), biochemical markers including lipid and lipoprotein fractions, fasting glucose, HbA1C, creatinine, CRP, Hcy, and angiotensin II in hypertensive patients. The results showed that age was positively correlated with Th1 (*P* = 0.005) and IL-17 (*P* = 0.008) levels but negatively correlated with the frequency of Th2 (*P* = 0.002) and IL-4 (*P* = 0.012); TC were positively correlated with IFN-*γ* (*P* = 0.029); LDL-C was negatively correlated with IL-4 (*P* = 0.020) and IL-17 (*P* = 0.016); and fasting glucose was positively correlated with Th1 (*P* = 0.019) and Th17 (*P* = 0.006) in hypertensive patients (Table 6). However, there were no significant correlations between Th1, Th2, and Th17 activities and years of hypertension, heart rate, BMI, HbA1C, creatinine, CRP, Hcy, or angiotensin II in these enrolled hypertensive patients.

To determine the independent predictors of the presence of nondipper hypertension, we performed binary logistic regression analyses. The results demonstrated that the frequencies of Th1 (OR 1.397, 95% CI 1.091 to 1.790; *P* = 0.008) and the frequencies of Th17 (OR 5.369, 95% CI 1.083 to 26.612; *P* = 0.040) were independently associated with the presence of the nondipper type. We also performed binary logistic regression analyses to determine the independent predictors of the presence of CAP. Only age (OR 1.168, 95% CI 1.062 to 1.285; *P* = 0.001) was independently associated with the presence of CAP in this study.

## 4. Discussion

It is well established that hypertension is a low-grade inflammatory disease [16, 17]. Inflammation not only promotes the rise of blood pressure but also accelerates the occurrence of target organ damage via the secretion of a great number of inflammatory cytokines and activating inflammatory cells such as macrophages, T cells, and dendritic cells [18, 19]. Clinical data demonstrated that inflammatory biomarkers such as CRP, IL-6, and TNF-*α* significantly increased in primary hypertension, and this increase is observed significantly more often in nondippers and is associated with the onset of target organ damage and cardiovascular events. Experimental studies have shown that blocking the IL-6 and TNF-*α* effect is helpful for controlling blood pressure and ameliorating target organ damage in hypertensive mice. Accumulating evidence has also demonstrated that Teff cells are critical inflammatory cells involved in a hypertension model and that regulating the activity of Teff cells may be a novel approach for preventing and treating hypertension and target organ damage [5, 7, 20–22]. However, the association between Teff cell activity and human hypertension remains uncertain.

In the present study, we investigated the circulating Th1, Th2, and Th17 levels in patients with primary hypertension. The results showed that the circulating Th1 and Th17 levels significantly increase and that the circulatingTh2 levels decreased in patients with hypertension. In addition, these immune disorders are particularly prominent in nondipper and hypertensive patients with CAP.

The pathogenic role of Th1 type response in hypertension was confirmed a decade ago. Using a rat hypertension model, Shao et al. found that angiotensin II infusion induced a Th1 type response and renal injury [20]. Although blood pressure was effectively ameliorated by olmesartan, which is an ARB, and hydralazine, which is a nonspecific vessel dilator, only olmesartan abolished Th1 type response and attenuated renal injury induced by exogenous angiotensin II. Mazzolai et al. demonstrated that the Th1 response induced by endogenous Ang II was associated with lesion unstable in atherosclerosis mice [5]. Our recent study also found same results in an exogenous angiotensin II-induced atherosclerotic model [6]. These studies indicate a close relationship between Th1 response and blood pressure elevation or organ damage during hypertension. Many studies have also demonstrated that the Th1 response is involved in blood pressure control. IFN-*γ* is the principal Th1 cytokine. Kamat et al. reported that IFN-*γ* deficiency significantly reduced blood pressure elevation induced by angiotensin II [22]. TNF-*α* is another important Th1 cytokine, while etanercept is a specific TNF-*α* antagonist. Elmarakby et al. found that etanercept abolished the effect of TNF-*α*, thereby ameliorating blood pressure in hypertensive rats [23, 24]. These studies suggest that the Th1 immune response plays a pathogenic role in a hypertension model. Furthermore, evidence from clinic investigations has showed that IFN-*γ* and TNF-*α* levels increased in hypertension patients compared with those in healthy subjects [12]. We also found that those patients with nondipper type or carotid atherosclerosis were associated with more excessive activation of the Th1 response. Taken together, these clinical and experimental studies suggest that blocking the Th1 effect may be a potential therapy for preventing and treating hypertension and its concurrent organ damage.

Our previous study did not find any difference in circulating Th2 levels between patients with coronary artery disease and control subjects [9], though we demonstrated a protective role for the Th2 response in an atherosclerotic model [6]. In the present study, we first found that Th2 levels decreased in patients with hypertension, particularly non-dippers patients and CAP patients. However, Th2 is not an independent predictor of the presence of nondippers and CAP. Interestingly, the role of IL-4 in clinical and experimental studies exhibited different results. Some clinical studies did not find a change in IL-4 levels in hypertension patients [12], while other studies found an increase in IL-4 levels [25], and some studies observed a decrease in IL-4 levels in patients with hypertension [26]. This contradiction was also observed in animal experimental studies. Peng et al. found that IL-4 deficiency has no effect on blood pressure values but significantly accelerates cardiac fibrotic remodeling in angiotensin II-induced hypertension [27]. Dicky et al. found that anti-IL-4 treatment resulted in a decrease in blood pressure in hypertensive NZBNZWF1 mice, suggesting a role of IL-4 in promoting blood pressure [28]. In contrast, Chatterjee et al. found that treatment with exogenous IL-4 significantly decreased blood pressure in P-PIC mice, which is a model of pregnancy-induced hypertension syndrome, indicating a protective role of IL-4 in hypertension [29]. Taken together, these studies suggested a complex role of the Th2 response in hypertension. A previous study has demonstrated that high Th2 and IL-4 levels are associated with a reduced risk of cardiovascular disease in healthy subjects, indicating a protective role of Th2 response in cardiovascular disease [30]. Whether the decrease in the Th2 response increases the risk of cardiovascular events in hypertension still remains unknown and should be investigated in the future.

The concept that the Th17 response plays a pathogenic role in hypertension has been widely accepted [7, 21, 22]. Madhur et al. first found that Th17 and IL-17 levels were significantly increased in angiotensin II-treated mice, accompanied by elevated blood pressure [7]. Using an IL-17 knockout model, they found that blood pressure sharply decreased and vascular dysfunction was abolished [7]. Furthermore, many studies have demonstrated that blocking the IL-17 effect protected mice from organ damage resulting from hypertension, indicating that upregulating the Th17 response was not only associated with increased blood pressure but also related to the onset of organ damage [21, 22]. In addition, Madhur et al. found that diabetic patients with hypertension have a higher level of serum IL-17 than diabetic patients without hypertension [7]. However, healthy subjects were not enrolled and circulating Th17 levels were not measured in this study [7]. Consistent with these results, many clinical studies have found that circulating IL-17 levels significantly increased in hypertensive patients [13, 14]. Moreover, Yao et al. found that serum IL-17 levels were significantly higher in subjects with prehypertension, defined as systolic BP of 120 to 139 mm Hg or diastolic BP of 80 to 89 mm Hg, than in subjects with optimal BP, defined as systolic BP < 120 mm Hg and diastolic BP < 80 mm Hg [31]. In the present study, we found that in addition to increased IL-17 levels, hypertensive patients had higher Th17 levels than healthy controls, especially those displaying the nondipper type. We also found that circulating Th17 levels were independently associated with the presence of the nondipper type but not CAP.

Tipton et al. found that male spontaneously hypertensive rats (SHR) have higher blood pressure and more CD4+ and Th17 cells in the kidney, whereas females have lower blood pressure and more T regulatory cells in the kidney, suggesting that different CD4+ T subsets play critical roles in different sexes in hypertension [32]. However, whether sex differences result in different effector T cell response in human hypertension has not yet been investigated. Therefore, we measured effector T cell activities in hypertension patients with different sexes. The results showed no differences in the levels of circulating effector T subsets or their functional cytokines between male and female hypertensive patients.

Data from previous studies revealed that medication use effectively regulates Teff cell activity [5, 6, 11, 14, 20]. Therefore, we analyzed the effect of prehospital medication on effector T cell activity. The results showed that patients receiving diuretics treatment displayed lower Th2 levels and higher Th17 levels than patients without diuretics treatment, while *β*-blocker use led to higher IL-17 levels than no use. We did not find significant effects for ACEI (ARB) or CCB use on effector T cell activity in hypertensive patients.

As noted above, Th1, Th2, and Th17 activities play a critical role in the hypertension model. However, whether the circulating Th1, Th2, and Th17 responses are associated with blood pressure values has yet been investigated. In the present study, a correlation analysis was performed to investigate the relationship between the circulating Th1, Th2, and Th17 responses and blood pressure in hypertensive patients. The results showed that IL-17 levels were negatively correlated with average DBP values in all hypertensive patients and nondippers but not in CAP patients. Moreover, IFN-*γ* levels were positively correlated with average night-time SBP values in all of the hypertensive patients. However, we did not see significant correlations between circulating Th1, Th2, and Th17 levels and blood pressure values in hypertensive patients and its subgroups.

There are some limitations in the present study. The sample size was too small for statistical significance in many contexts to be established. Therefore, the sample size should be increased in subsequent studies. Second, a prospective interventional study is suitable for investigating whether antihypertensive medication use will effectively reduce blood pressure and regulate the activity of Teff cells.

In conclusion, the circulating Th1, Th2, and Th17 levels changed in hypertensive patients, particularly in nondippers and CAP patients. Additionally, Th1 and Th17 were independently associated with the nondipper type, suggesting a close relationship between Th1 and Th17 responses and nondipper hypertension. Regarding the evidence from clinical and experimental studies, regulating Teff activity may be a novel therapy for preventing and treating hypertension and target organ damage.

## Figures and Tables

**Table 1:** Clinical characteristics of patients.

Characteristics	Control (*n* = 15)	HT (*n* = 45)	Dipper (*n* = 20)	Nondipper (*n* = 25)	NCAP (*n* = 28)	CAP (*n* = 17)
Age (years)	47.7 ± 9.0	46.4 ± 13.5	44.2 ± 16.7	48.1 ± 10.3	40.0 ± 10.1	56.9 ± 11.9∗
Sex (male/female)	7/8	27/18	11/9	16/9	16/9	16/9
Year of hypertension (years)	—	10.2 ± 9.2	11.8 ± 10.7	8.9 ± 7.9	6.6 ± 6.2	16.1 ± 10.5
Office SBP (mm Hg)	112 ± 9.0	143 ± 19^∗^	144 ± 17^∗^	143 ± 20^∗^	144 ± 19^∗^	142 ± 18^∗^
Office DBP (mm Hg)	74 ± 9.0	92 ± 16^∗^	93 ± 15^∗^	91 ± 17^∗^	96 ± 15^∗^	85 ± 15
Heart rate (beats/min)	67 ± 6	70 ± 8	71 ± 9	69 ± 7	71 ± 6	67 ± 10
BMI (kg/m^2^)	23.11 ± 2.12	26.79 ± 3.58^∗^	26.97 ± 3.50^∗^	26.64 ± 3.71^∗^	26.68 ± 4.14^∗^	26.96 ± 2.51^∗^
TG (mmol/L)	1.71 ± 1.56	1.98 ± 2.18	1.96 ± 1.49	2.00 ± 2.64	1.84 ± 1.59	2.20 ± 2.95
TC (mmol/L)	4.42 ± 0.74	4.62 ± 0.74	4.66 ± 0.74	4.59 ± 0.76	4.51 ± 0.57	4.79 ± 0.96
LDL-C (mmol/L)	2.47 ± 0.73	2.84 ± 0.62	2.91 ± 0.66	2.79 ± 0.6	2.79 ± 0.65	2.93 ± 0.57
HDL-C (mmol/L)	1.25 ± 0.3	1.06 ± 0.26^∗^	1.07 ± 0.25	1.05 ± 0.28	1.04 ± 0.27	1.08 ± 0.24
GLU (mmol/L)	5.37 ± 0.35	5.33 ± 0.65	5.23 ± 0.57	5.41 ± 0.71	5.18 ± 0.62	5.59 ± 0.64
HbA1c (%)	5.37 ± 0.39	5.47 ± 0.51	5.37 ± 0.4	5.55 ± 0.58	5.32 ± 0.49	5.71 ± 0.46
Creatinine (*μ*mol/L)	71.33 ± 14.48	77.42 ± 32.11	73.06 ± 18.88	80.92 ± 39.75	79.12 ± 38.24	74.62 ± 18.83
CRP (mg/L)	0.64 ± 0.77	2.42 ± 3.53^∗^	1.77 ± 1.95	2.94 ± 4.39	2.23 ± 3.73	2.73 ± 3.27
Hcy (pg/ml)	11.27 ± 4.75	16.38 ± 7.14^∗^	18.05 ± 9.18^∗^	15.04 ± 4.76	17.7 ± 8.31^∗^	14.20 ± 3.96
Ang II (pg/ml)	37.71 ± 8.69	69.61 ± 35.39^∗^	72.65 ± 44.08^∗^	67.17 ± 27.28^∗^	69.54 ± 40.90^∗^	69.72 ± 24.93^∗^
Medical treatments, *n* (%)						
ACEI/ARB	—	30 (66.7)	14 (70)	16 (64)	15 (53.6)	15 (88.2)
*β*-Blocker	—	24 (53.3)	10 (50)	14 (56)	15 (53.6)	9 (52.9)
CCB	—	33 (73.3)	14 (70)	19 (76)	19 (67.9)	14 (82.3)
Diuretics	—	11 (24.4)	2 (10)	9 (36)	4 (14.3)	7 (41.2)

Note: the data are given as the mean ± SD or number of patients. SBP: systolic blood pressure; DBP: diastolic blood pressure; BMI: body mass index; TG: total triglycerides; TC: total cholesterol; HDL-C: high-density lipoprotein cholesterol; LDL-C: low-density lipoprotein cholesterol; GLU: fasting glucose; CRP: C-reactive protein; Hcy: homocysteine; Ang II: angiotensin II; ACEI: angiotensin-converting enzyme inhibitor; ARB: angiotensin receptor blocker; CCB: calcium channel blocker. ^∗^*P* < 0.05 versus control.

**Table 2:** Data from ABPM of patients.

	Control (*n* = 15)	HT (*n* = 45)	Dipper (*n* = 20)	Nondipper (*n* = 25)	NCAP (*n* = 28)	CAP (*n* = 17)
Average 24 h SBP	109 ± 8	134 ± 15^∗^	132 ± 16^∗^	135 ± 15^∗^	137 ± 15^∗^	128 ± 14^∗^^,#^
Average 24 h DBP	66 ± 6	86 ± 13^∗^	85 ± 11^∗^	87 ± 15^∗^	90 ± 10^∗^	78 ± 14^∗^^,#^
Average daytime SBP	116 ± 7	136 ± 15^∗^	137 ± 16^∗^	136 ± 15^∗^	140 ± 15^∗^	129 ± 13^∗^^,#^
Average daytime DBP	70 ± 5	89 ± 14^∗^	89 ± 13^∗^	89 ± 15^∗^	93 ± 11^∗^	81 ± 15^∗^^,#^
Average night-time SBP	102 ± 9	128 ± 17^∗^	123 ± 17^∗^	133 ± 16^∗^	130 ± 17^∗^	125 ± 18^∗^
Average night-time DBP	62 ± 5	81 ± 13^∗^	77 ± 10^∗^	84 ± 14^∗^	85 ± 11^∗^	75 ± 15^∗^^,#^

Note: the data are given as the mean ± SD. SBP: systolic blood pressure; DBP: diastolic blood pressure. ^∗^*P* < 0.05 versus control; ^#^*P* < 0.05 CAP versus NCAP.

**Table 3:** The levels of circulating Th1, Th2, and Th17 cells and their functional cytokines in patients.

	Control (*n* = 15)	HT (*n* = 45)	Dipper (*n* = 20)	Nondipper (*n* = 25)	NCAP (*n* = 28)	CAP (*n* = 17)
Th1 (%)	16.28 ± 2.99	24.89 ± 3.88^∗∗^	22.54 ± 3.20^∗∗^	26.77 ± 3.34^∗∗^^,§§^	23.83 ± 3.90^∗∗^	26.63 ± 3.18^∗∗^^,#^
Th2 (%)	3.24 ± 0.54	1.47 ± 0.45^∗∗^	1.66 ± 0.44^∗∗^	1.31 ± 0.40^∗∗^^,§^	1.62 ± 0.43^∗∗^	1.22 ± 0.37^∗∗^^,#^
Th17 (%)	0.88 ± 0.27	1.49 ± 0.56^∗∗^	1.20 ± 0.46	1.72 ± 0.52^∗∗^^,§§^	1.48 ± 0.61^∗∗^	1.69 ± 0.64^∗∗^
IFN-*γ* (pg/mL)	12.56 ± 2.76	18.20 ± 4.46^∗∗^	16.43 ± 3.94^∗^	19.62 ± 4.42^∗∗^^,§^	17.91 ± 3.94^∗∗^	18.68 ± 5.31^∗∗^
IL-4 (pg/mL)	28.31 ± 9.15	21.46 ± 7.57^∗∗^	23.12 ± 7.46	20.14 ± 7.54^∗∗^	23.22 ± 7.79	18.57 ± 6.40^∗∗^
IL-17 (pg/mL)	5.84 ± 1.99	8.16 ± 2.35^∗∗^	7.37 ± 2.32	8.79 ± 2.22^∗∗^	7.68 ± 2.15^∗^	8.94 ± 2.51^∗∗^

Note: the data are given as the mean ± SD. ^∗^*P* < 0.05 versus control, ^∗∗^*P* < 0.01 versus control, ^§^*P* < 0.05 nondipper versus dipper, ^§§^*P* < 0.01 nondipper versus dipper, and ^#^*P* < 0.05 CAP versus NCAP.

**Table 4:** Circulating Th1, Th2, and Th17 levels according to sex and medication in hypertensive patients.

	Th1 (%)	Th2 (%)	Th17 (%)	IFN-*γ* (pg/mL)	IL-4 (pg/mL)	IL-17 (pg/mL)
Sex						
Male (*n* = 27)	24.48 ± 3.71	1.48 ± 0.45	1.52 ± 0.56	17.92 ± 3.59	21.40 ± 7.77	8.30 ± 2.46
Female (*n* = 18)	25.50 ± 4.15	1.45 ± 0.46	1.44 ± 0.56	18.62 ± 5.62	21.56 ± 7.47	7.93 ± 2.22
ACEI/ARB						
Yes (*n* = 30)	24.92 ± 3.98	1.39 ± 0.46	1.57 ± 0.58	18.16 ± 4.73	21.47 ± 7.88	8.24 ± 2.38
No (*n* = 15)	24.83 ± 3.80	1.62 ± 0.39	1.32 ± 0.48	18.29 ± 4.03	21.46 ± 7.16	7.99 ± 2.35
*β*-Blocker						
Yes (*n* = 24)	25.13 ± 4.18	1.49 ± 0.41	1.58 ± 0.59	19.20 ± 4.43	22.56 ± 7.70	8.86 ± 2.43∗
No (*n* = 21)	24.61 ± 3.58	1.44 ± 0.50	1.39 ± 0.51	17.73 ± 4.40	20.21 ± 7.40	7.35 ± 2.01
CCB						
Yes (*n* = 33)	24.95 ± 3.97	1.44 ± 0.39	1.50 ± 0.58	17.47 ± 4.52	21.50 ± 7.36	8.50 ± 2.42
No (*n* = 12)	24.71 ± 3.78	1.54 ± 0.59	1.45 ± 0.50	20.20 ± 3.77	21.38 ± 8.45	7.19 ± 1.91
Diuretics						
Yes (*n* = 11)	26.64 ± 3.29	1.15 ± 0.29^*^	1.86 ± 0.59^*^	19.53 ± 4.96	18.74 ± 7.26	9.11 ± 1.99
No (*n* = 34)	24.32 ± 3.93	1.57 ± 0.44	1.37 ± 0.50	17.77 ± 4.28	22.35 ± 7.56	7.85 ± 2.40

Note: the data are given as the mean ± SD. ACEI: angiotensin-converting enzyme inhibitor; ARB: angiotensin receptor blocker; CCB: calcium channel blocker. ^∗^*P* < 0.05.

**Table 5:** Correlation analysis between circulating Th1, Th2, and Th17 levels and blood pressure in hypertensive patients.

	Th1 (%)	Th2 (%)	Th17 (%)	IFN-*γ* (pg/mL)	IL-4 (pg/mL)	IL-17 (pg/mL)
HT (*N* = 45)						
Average 24 h SBP	0.052	0.128	−0.033	0.269	0.104	−0.144
Average 24 h DBP	−0.089	0.231	−0.133	0.089	0.058	0.284
Average daytime SBP	−0.093	0.254	−0.172	0.184	0.192	−0.221
Average daytime DBP	−0.117	0.202	−0.191	0.101	0.173	−0.336^∗^
Average night-time SBP	0.272	−0.026	0.166	0.363^∗^	−0.079	0.005
Average night-time DBP	0.148	0.097	−0.066	0.267	0.004	−0.221
Nondipper (*n* = 25)						
Average 24 h SBP	0.051	0.238	−0.193	0.274	0.032	−0.269
Average 24 h DBP	−0.181	0.424	−0.291	0.070	0.039	−0.392^∗^
Average daytime SBP	−0.012	0.291	−0.25	0.205	0.105	−0.293
Average daytime DBP	−0.169	0.369	−0.349	0.105	0.177	−0.425^∗^
Average night-time SBP	0.120	0.252	−0.151	0.286	−0.094	−0.208
Average night-time DBP	−0.085	0.397	−0.347	0.178	0.098	−0.385^∗^
CA (*n* = 17)						
Average 24 h SBP	0.429	−0.198	0.151	0.380	0.069	−0.027
Average 24 h DBP	0.163	−0.050	−0.011	0.188	−0.024	−0.246
Average daytime SBP	0.078	−0.158	0.078	0.286	0.139	−0.049
Average daytime DBP	0.140	−0.109	−0.102	0.207	0.179	−0.301
Average night-time SBP	0.558	−0.178	0.213	0.443	−0.035	0.011
Average night-time DBP	0.349	0.142	−0.056	0.370	0.118	−0.220

SBP: systolic blood pressure; DBP: diastolic blood pressure. ^∗^*P* < 0.05.

**Table 6:** Correlation analysis between circulating Th1, Th2, and Th17 levels and clinical parameters in hypertensive patients.

	Th1 (%)	Th2 (%)	Th17 (%)	IFN-*γ* (pg/mL)	IL-4 (pg/mL)	IL-17 (pg/mL)
Age (years)	0.408^∗∗^	−0.448^∗∗^	0.127	0.121	−0.372^∗^	0.389^∗∗^
Year of hypertension (years)	0.143	−0.181	−0.051	0.010	−0.218	0.018
Heart rate (beats/min)	−0.111	0.209	−0.094	0.026	0.234	−0.019
BMI (kg/m^2^)	0.028	0.185	0.037	0.098	−0.124	−0.062
TG (mmol/L)	0.199	−0.076	0.000	0.246	0.060	0.096
TC (mmol/L)	0.090	−0.110	−0.226	0.325^∗^	−0.266	−0.140
LDL-C (mmol/L)	0.021	−0.011	−0.174	0.198	−0.346^∗^	−0.358^∗^
HDL-C (mmol/L)	−0.189	−0.113	−0.097	−0.173	0.101	0.065
GLU (mmol/L)	0.348^∗^	−0.160	0.405^∗^	0.075	−0.209	0.238
HbA1c (%)	0.256	−0.207	0.175	−0.039	−0.196	0.190
Creatinine (*μ*mol/L)	0.174	−0.128	0.065	0.175	−0.129	0.034
CRP (mg/L)	0.235	−0.116	0.163	0.255	−0.109	0.277
Hcy (pg/mL)	−0.253	0.257	−0.143	−0.168	0.044	−0.089
Ang II (pg/mL)	−0.091	−0.053	0.137	−0.103	0.041	0.148

BMI: body mass index; TG: total triglycerides; TC: total cholesterol; HDL-C: high-density lipoprotein cholesterol; LDL-C: low-density lipoprotein cholesterol; GLU: fasting glucose; CRP: C-reactive protein; Hcy: homocysteine; Ang II: angiotensin II. ^∗^*P* < 0.05 and ^∗∗^*P* < 0.01.
